# In-vivo distortion of through-plane flow by spiral phase-contrast imaging

**DOI:** 10.1186/1532-429X-14-S1-W61

**Published:** 2012-02-01

**Authors:** Iain T Pierce, Peter D Gatehouse, David N Firmin

**Affiliations:** 1 NHLI, Imperial College London, London, UK; 2 CMR Unit, Royal Brompton Hospital, London, UK

## Summary

To explain previously unrecognised consequences of two sources of phase curvature over the vessel cross-section in spiral imaging i.e. off-resonance and the velocity-encoded phase-shift.

## Background

The effects of off-resonance frequency errors during spiral readouts [Yudilevich and Stark, 1987] are known. Here we explain previously unrecognised intra-voxel dephasing consequences of two sources of phase curvature over the vessel cross-section in through-plane flow imaging using spirals i.e. off-resonance and the velocity-encoded phase-shift, including the consequences for in-vivo measurements.

## Methods

Laminar through-plane flow phantom (50cm/s) and popliteal artery studies were acquired at Venc=50cm/s with initial off-resonance offsets ± 0 10, 20, 40Hz representing <1ppm at 1.5T. Reference (“Ref”) and velocity-encoded (“Vel”) magnitude and phase images were obtained (as a cine in-vivo). Spiral FOV was 150 mm, 1mm resolution, duration 25.7ms; TE/TR 4.0/32.7 ms, FA 30°, 4 interleaves.

## Results

**Figure 1:**
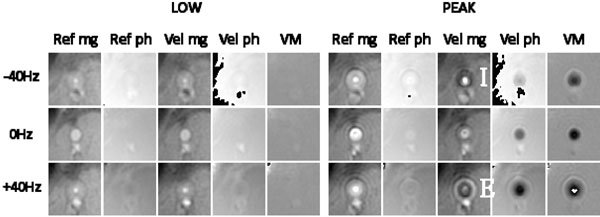
Reference, Ref and Velocity encoded, Vel magnitude and phase images with corresponding velocity maps of the popliteal artery at low and peak velocity at 50cm/s VENC with initial frequency offsets -40, 0, +40 Hz. Low velocity images show symmetric distortion of the artery on ref. and vel. magnitude images while the phase images show a positive phase slope with negative off-resonance and negative phase slope with positive off-resonance; and is similar between ref. and vel. images. Peak velocity shows similar distortions on the ref. magnitude and phase images as at low velocity. The vel. magnitude images show an implosion (I) and explosion (E) effect as the signal loss due to shear phase dispersion is increased and decreased respectively; the vel. phase images show the off-resonance induced phase adding destructively and constructively to the vel. induced phase resulting in varying velocity profiles across the artery in the VMs.

Figure 1 shows magnitude and phase images for Ref and Vel scans with corresponding velocity maps (VM). For low flow rates the off-resonance blurring at +40Hz and -40Hz is similar for both Ref. and Vel. images. However, high velocities cause an apparent “implosion” (Figure 1-I) and “explosion” (Figure 1-E) of the vessel for opposite off-resonance frequencies. From the distorted VMs, peak velocity was measured at 37.8, 48.6, 56.3cm/s for -40, 0, 40Hz off-resonance respectively.

**Figure 2:**
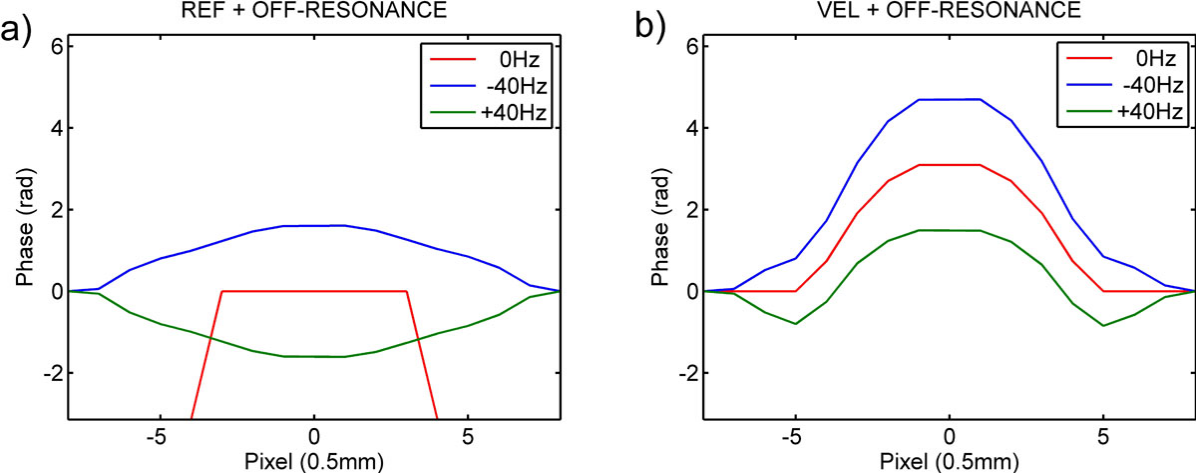
The phase across a small vessel with no flow is ideally flat with infinitely steep sides (a-red); off-resonance causes a distortion of the flat phase such that it becomes curved (simulated off-resonance) (a-blue/green) and can be seen in the LOW flow phase images in Figure 2 where both Ref ph and Vel ph are similar. Velocity mapping induces phase relative to velocity within a pixel, the ideal Ref. phase over the vessel with laminar flow remains flat (a-red) and for Vel. phase has parabolic profile (b-red). When the Vel. image is acquired with off-resonance, the phase from these 2 effects can add constructively or destructively resulting in distortion of the velocity profile (Figure 1-PEAK VM). Signal loss in edge pixels with high velocity shear can be increased (b-blue) or decreased (b-green) depending on the direction of the off-resonance phase resulting in the implosion (Figure 1-I) and explosion (Figure 1-E) artefacts in Vel. mg images.

For theoretical explanation, Figure 2a) depicts the ideal diametrical phase line profile across a tube velocity-compensated spiral imaging, red line = on-resonance and blue/green lines = phase curvature induced by ±40Hz off-resonance error. Figure 2b)-red shows velocity-encoded phase over the vessel with parabolic flow. Figure 2b)-blue shows the consequence of their addition: the increased radial slope worsens intra-voxel dephasing in all but the central pixels of a laminar flow (“implosion” effect, Figure 1-I). In Figure 2b)-green the opposing phase curvatures lead to a cancellation of the radial phase slope at some radius, which can lie outside the true lumen (“explosion” effect, Figure 1-E).

## Conclusions

The distortion of the velocity distribution over the in-vivo vessel distorts peak velocity by ~20% at -40Hz off-resonance at these sequence parameters. Separate tests eliminated through-plane gradient fields as a cause, including eddy-current effects after the velocity-encoding pulses.

These effects on magnitude images and velocity distributions at <1ppm off-resonance are potentially difficult for >20ms spiral readouts in small vessel applications at least, perhaps more so near B0-distortions such as lungs. Shorter spirals and avoiding large intra-voxel radial phase shear are some-what incompatible with rapid flow work.

